# Advancements in Diagnostic and Therapeutic Interventions of Non-alcoholic Fatty Liver Disease: A Literature Review

**DOI:** 10.7759/cureus.44924

**Published:** 2023-09-08

**Authors:** Ahtshamullah Chaudhry, Jawad Noor, Saima Batool, Ghulam Fatima, Riwad Noor

**Affiliations:** 1 Internal Medicine, St. Dominic Hospital, Jackson, USA; 2 Pathology, Nishtar Medical University, Multan, PAK; 3 Internal Medicine, Medical Unit, Abbasi Shaheed Hospital, Karachi, PAK; 4 Public Health, Nishtar Hospital, Multan, PAK

**Keywords:** therapeutic, diagnostic, innovations, non-alcoholic steatohepatitis, non-alcoholic fatty liver disease

## Abstract

Non-alcoholic fatty liver disease (NAFLD) is one of the most common diseases of the liver globally. Non-alcoholic steatohepatitis (NASH) has a complicated pathophysiology which includes lipid buildup, oxidative stress, endoplasmic reticulum stress, and lipotoxicity. Recently, there has been tremendous improvement in understanding of NASH pathogenesis due to advancements in the scientific field. It is being investigated how non-invasive circulating and imaging biomarkers can help in NAFLD and NASH diagnosis and monitoring the progress. Multiple medications are now undergoing clinical trials for the treatment of NASH, and lifestyle changes have been acknowledged as one of the main treatment methods. The purpose of this review article is to discuss the incidence of NAFLD globally, management issues with NASH, and its relation to the metabolic syndrome. It explains pathophysiology as well as therapeutic strategies using natural items, dietary changes, and pharmaceutical treatments. While emphasizing the necessity for surrogate endpoints to facilitate medication development for NASH, the study also considers the potential of non-invasive imaging biomarkers including magnetic resonance imaging (MRI) and magnetic resonance elastography (MRE).

## Introduction and background

An abnormality in the metabolism of hepatic fatty acids (FA) results in the development of non-alcoholic fatty liver disease (NAFLD). Non-alcoholic steatohepatitis (NASH) and non-alcoholic fatty liver (NAFL) are the two main kinds. While NASH is characterized by steatosis combined with inflammation, hepatocellular damage, lobular inflammation, and fibrosis, NAFL refers to the presence of fat in the liver without considerable inflammation [1]. Insulin resistance (IR)-related regulation of lipolysis at the level of adipose tissue plays a major role in developing NAFLD [2]. 

The accumulation of FA in the liver is facilitated by the over-expression of CD36 fatty acid translocase and adipocyte fatty acid-binding proteins (FABP), particularly FABP-4 and FABP-5 [3]. De novo lipogenesis (DNL), a detoxification activity that formulates new FA from excess glucose, is also a significant contributor to hepatic lipid accumulation in NAFLD. The activation of two transcription factors, sterol regulatory element-binding protein-1c (SREBP-1c) and carbohydrate-responsive element-binding protein (ChREBP), plays a vital part in the upregulation of hepatic DNL [4]. Additionally, a small amount of the FA pool in NAFLD is derived from dietary triglycerides associated with chylomicrons [5]. 

A recent increase in metabolic syndrome and its associated conditions like visceral obesity, diabetes mellitus type 2, and dyslipidemia has caused an increase in the incidence of NAFLD. This syndrome raises death rates as well as the risk of developing cardiovascular disorders [1,4]. Furthermore, NAFLD has been linked to liver cancer. Therefore, early detection and timely treatment of NAFLD is crucial [5]. This paper provides a comprehensive overview of the advancements in therapeutic techniques and diagnostic approaches for NAFLD, highlighting their evolution over time.

## Review

Pathogenesis of NAFLD

The pathogenesis of NAFLD involves a "two-hit" hypothesis. The "first hit" is insulin resistance, which leads to excessive FA flow into the liver. The "second hit" is inflammation attributed to gut-derived endotoxin, oxidative stress, and mitochondrial dysfunction [3]. Oxidation occurs due to many factors, such as cytokine injury, hyperinsulinemia, changes in the function of the immune system, and energy homeostasis. According to the studies conducted by Anstee et al. in 2013 and Vetrano et al. in 2023, several factors contribute to the development of NASH, which is characterized by excessive accumulation of cholesterol, inflammation, liver cell injury, and hepatocyte cell death. These factors can lead to liver disease and the development of hepatocellular carcinoma (HCC), a type of liver cancer [2, 6]. 

**Figure 1:**
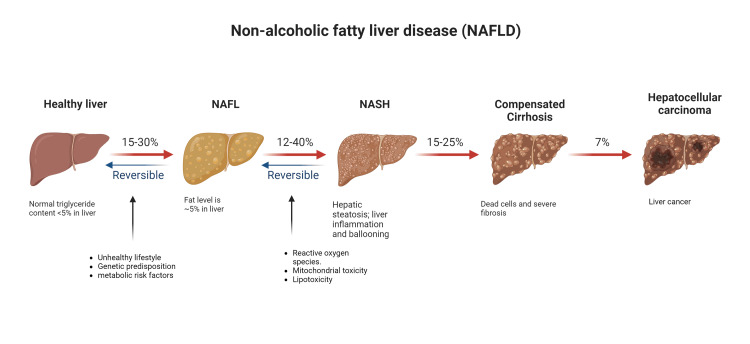
Different stages of NAFLD The figure depicts the stages of NAFLD, from healthy liver to NAFL that progresses to NASH. The disease progression leads to cirrhosis and hepatocellular carcinoma (HCC). NAFL: Non-alcoholic fatty liver, NASH: Non-alcoholic steatohepatitis Figure created by the authors with BioRender.com.

Recent research, as highlighted by Cholankeril et al. [7], has demonstrated that HCC can also arise in non-cirrhotic patients with NASH. The degree of fibrosis may play a crucial role in determining the future risk of HCC in the absence of cirrhosis, as evidenced by studies conducted by [1, 8]. Specifically, patients with NASH and advanced fibrosis have been found to face a heightened HCC risk. Figure 1 explains the development of NAF below.

The pathogenesis of NAFLD involves several interconnected mechanisms that contribute to its development and progression.

Lipid Accumulation

One of the primary mechanisms is lipid accumulation. When the intake of energy exceeds energy expenditure, excess energy is stored as lipids, leading to the buildup of triglycerides in hepatocytes. This lipid accumulation arises from multiple sources, including white adipose tissue, de novo lipogenesis, and a high-fat and/or high-sugar diet [9]. Such excess triglyceride synthesis contributes to the manifestation of hepatic steatosis, characterized by the accumulation of fat in the liver [3].

Oxidative Stress

Another critical mechanism is oxidative stress. In NAFLD, an overabundance of fatty acids compromises mitochondrial function and beta-oxidation, resulting in mitochondrial dysfunction. This dysfunctional state gives rise to the production of reactive oxygen species (ROS), which are highly reactive molecules. ROS, in turn, induces oxidative stress, triggering inflammation and causing damage to hepatocytes. The interplay between lipid accumulation and oxidative stress creates a vicious cycle, exacerbating liver injury [10].

Endoplasmic Reticulum (ER) Stress

Endoplasmic reticulum (ER) stress is also closely associated with NAFLD. The ER is responsible for protein synthesis, folding, and quality control. Disrupted ER homeostasis leads to the accumulation of unfolded or misfolded proteins, triggering ER stress. Initially, the unfolded protein response (UPR) is activated to restore protein homeostasis. However, if the UPR fails to promote cell survival, proapoptotic ER stress pathways are activated, resulting in cell death. The presence of ER stress further contributes to the progression of NAFLD [11].

Lipotoxicity

Additionally, lipotoxicity plays a significant role in the pathogenesis of NAFLD. Lipotoxicity refers to the toxic effects caused by sustained high concentrations of lipids and metabolites in non-adipose tissues. In NAFLD, lipotoxic substances accumulate in hepatocytes, leading to liver damage. Insulin resistance, increased plasma free fatty acids (FFAs), mitochondrial dysfunction, oxidative stress, ER stress, and inflammatory responses collectively contribute to the lipotoxicity observed in NAFLD [9].

Management approaches for NAFLD

Natural products and lifestyle modifications are being explored as potential therapeutic options for NAFLD, as there are currently no FDA-approved drugs specifically for its treatment. These natural products can target various aspects of NAFLD pathogenesis, including lipid metabolism, oxidative stress, ER stress, and inflammation. The therapeutic techniques to treat NAFLD are mainly focused on inflammation, fibrosis, and hepatic steatosis because the pathogenesis of NAFLD is complex [12-14].

In terms of lipid metabolism, certain natural products have shown promise by modulating the adenosine monophosphate-activated protein kinase (AMPK) pathway. Examples include antrodan from Antrodia cinnamomea, emodin from Radix Polygoni Multiflori, and flavonoids from Lomatogonium rotatum [15].

Oxidative stress is a crucial factor in NAFLD, and natural products with antioxidant properties have been investigated. Hesperetin from citrus fruits, as well as Gastrodin, yellow loosestrife, geniposide, xyloketal B, chicory seed extract, Crataegus azarolus var. aronia, apigenin, scutellarin, and alpinetin have demonstrated antioxidant effects and the ability to regulate lipid metabolism through pathways such as nuclear factor erythroid-derived 2-like 2 (Nrf2) or peroxisome proliferator-activated receptor (PPAR) activation [10].

ER stress is closely associated with lipid accumulation and liver injury in NAFLD, and certain natural products have shown potential in alleviating ER stress. Coffee, Amomum villosum var. xanthioides, Eucommia ulmoides Oliver leaves, aucubin, geniposide, Ixeris dentata, and tanshinone IIA have demonstrated the ability to mitigate ER stress and related liver injury.

Inflammation plays a critical role in NAFLD progression, and natural products with anti-inflammatory properties have been studied. Resveratrol from grapes and red wine, Cynanchum atratum, Lycopus lucidus, Atractylodes macrocephala, and Salvianolic acid A have shown anti-inflammatory effects through mechanisms like AMPK activation [3-15].

Lifestyle Interventions and Mediterranean Diet in NAFLD

In addition to natural products, lifestyle interventions play a crucial role in improving the symptoms and signs of NAFLD. Weight loss and lifestyle changes, such as adopting a balanced diet, regular physical activity, and avoiding alcohol consumption, are the main approaches to improving outcomes of NAFLD [12]. The Mediterranean diet (MD) includes food rich in macronutrients that are helpful in modulating glycosidic and lipid metabolism and thus help with fatty liver disease. MD consists of 30-35% fat which comes from consuming extra virgin olive oil, nuts, and omega-3 containing foods (provides mono-unsaturated fatty acids (MUFAs) and poly-unsaturated fatty acids (PUFAs)), 25-30% of protein which comes from vegetable sources and 40-45% carbohydrates (50-70% of that carbohydrates should come from low glycemic index and high fibers [16]. The combination of all these effects visceral obesity, dyslipidemia, insulin resistance, and chronic inflammation which improves metabolic syndrome leading to improvement in NAFLD [16]. Multiple studies have shown that MD has anti-inflammatory and antioxidant properties which decrease the progression of NAFLD. These benefits are due to the nutraceutical effect of bioactive compounds and phytochemicals like fibers MUFAs, phytosterols, and omega-3 fatty acids [17]. MD diet also affects gut-microbiota production which also affects metabolic syndrome and NAFLD. Furthermore, between the 1950s and 1980s, Ancel Keys published multiple studies that showed improvement in cardiovascular and cancer mortality in people from Greece and Italy and their diet mostly was MD [17]. 

**Figure 2:**
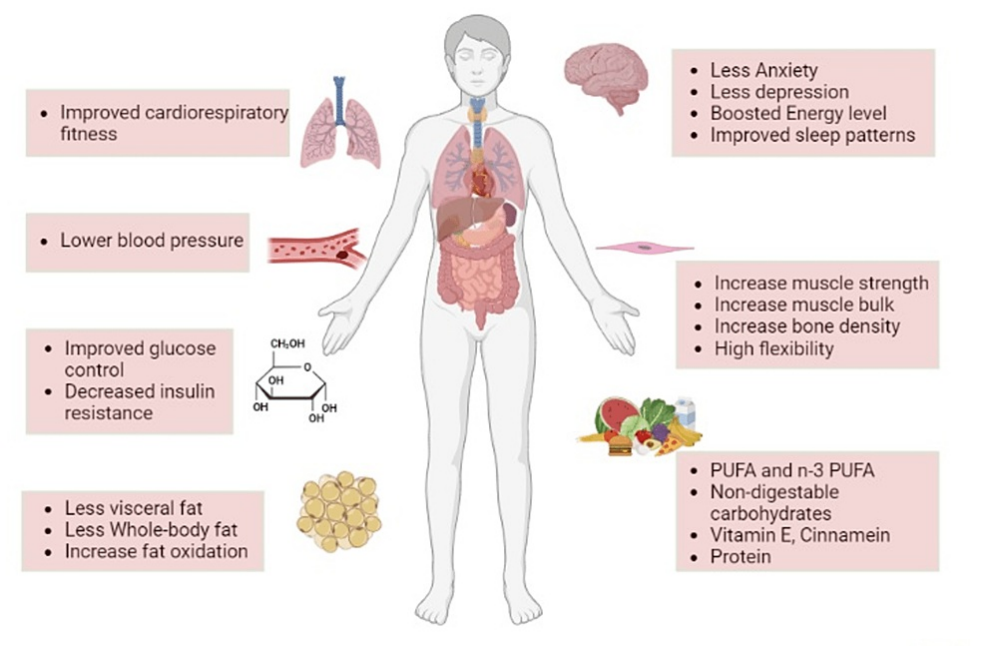
Lifestyle interventions in NAFLD/NASH The figure depicts lifestyle interventions in NAFLD/NASH, including improved brain functioning, muscle strength, volunteering exercise leading to respiratory fitness, less body fat, and improved diet. NAFLD: Non-alcoholic fatty liver disease, NASH: Non-alcoholic steatohepatitis; PUFA: poly-unsaturated fatty acid Figure created by the authors with BioRender.com.

Figure 2 shows lifestyle interventions in NAFLD/NASH:

Lifestyle modifications are particularly important in patients with diabetes, obesity, and metabolic syndrome, as these conditions frequently coexist with NAFLD and increase cardiovascular risks. Fatty liver disease encompasses a spectrum of hepatic pathology, ranging from simple steatosis to non-alcoholic steatohepatitis, cirrhosis, hepatocellular carcinoma, and end-stage liver disease. The most recent guidelines suggest the management and treatment of patients with NAFLD considering both the liver disease and the associated metabolic co-morbidities. Diet and physical exercise are considered the first line of treatment for patients with NAFLD, but their results on therapeutic efficacy are often contrasting. Behavioral therapy is necessary most of the time to achieve a sufficient result [15, 18-20].

Pharmacological Therapy for NAFLD/NASH

Since the last decade, there has been an increase in pharmacological therapies in developing drugs treating NASH [21]. Statins (lipid-lowering agents), which are primarily used to reduce cardiovascular risk, have been found to be beneficial in treating patients with NAFLD, even in cases where the disease has progressed to non-alcoholic steatohepatitis (NASH) [4, 22]. Other non-statin hypolipidemic therapies, such as ezetimibe, bile acid sequestrants, PCSK9 inhibitors, and omega-3 fatty acids, may also confer liver benefits and reduce residual lipid risks in patients with NAFLD and NASH [23, 24].

Several antihyperglycemic drugs have shown promise in treating NAFLD/NASH, including pioglitazone, sitagliptin, GLP-1 receptor agonists, and SGLT2 inhibitors [21, 25]. Insulin sensitizers such as pioglitazone and high-dose vitamin E have been reported to improve the histology of patients with NASH. However, it's important to note that not all pharmacological interventions have been effective in improving liver histology in patients with NAFLD, such as metformin and ursodeoxycholic acid (UDCA). Liver biopsy is currently considered the gold standard for the diagnosis and staging of NAFLD because of the absence of noninvasive and specific biomarkers. Personalized medicine approaches and targeted therapies addressing the underlying mechanisms of NAFLD are also being explored [11, 22]. Table 1 shows the pharmacological interventions in NAFLD.

**Table 1:** Pharmacological interventions for NAFLD, their mechanisms and possible limitations. Adapted from Negi et al. [22] NAFLD: Non-alcoholic fatty liver disease

Pharmacological Intervention	Mechanism of Action	Limitations
Pioglitazone	Improves insulin sensitivity, reduces liver inflammation	Weight gain, fluid retention, increased risk of heart failure
Vitamin E	Antioxidant properties, reduces oxidative stress in the liver	High doses may increase the risk of hemorrhagic stroke
Ursodeoxycholic acid (UDCA)	Modulates bile acid metabolism, reduces liver inflammation	Limited evidence of effectiveness, variable response among patients
Omega-3 fatty acids	Anti-inflammatory effects, improves lipid metabolism	High doses may increase the risk of bleeding, gastrointestinal side effects
Metformin	Improves insulin sensitivity, reduces glucose production in the liver	Gastrointestinal side effects, lactic acidosis (rare but serious complication)
Statins	Reduces cholesterol levels, may have anti-inflammatory effects	Muscle pain, liver toxicity, potential drug interactions
Fibrates	Lowers triglyceride levels, may improve liver steatosis	Gastrointestinal side effects, increased risk of gallstones
Pentoxifylline	Reduces inflammation and fibrosis in the liver	Gastrointestinal side effects, limited evidence of effectiveness
Vitamin D	Modulates immune response, may reduce liver inflammation	Limited evidence of effectiveness, potential for vitamin D toxicity
Antioxidant supplements	Neutralize oxidative stress, protect liver cells	Limited evidence of effectiveness, potential for adverse effects in high doses

Advancement in the Therapeutic Strategies and Drugs

Managing patients with NAFLD involves addressing the disease stage and risk factors. Key strategies include lifestyle modification, targeting metabolic syndrome, managing cirrhosis complications, and pharmacotherapy for high-risk patients. Lifestyle modifications aim to reduce obesity, increase physical activity, and manage metabolic risk factors. Aggressive lifestyle modification is recommended for patients with severe steatohepatitis and fibrosis. Cirrhosis patients require HCC surveillance and treatments to reduce HCC risk. Therapeutic techniques target inflammation, fibrosis, and hepatic steatosis, focusing on weight loss, lipid metabolism improvement, cardiovascular risk reduction, and insulin sensitivity enhancement [11].

Monitoring the progression and identification advancement of disease in NAFLD/NASH 

Circulating Biomarkers of NAFLD/NASH

To monitor the effectiveness of NAFLD treatment and assess disease progression, several biomarkers can be used. Serum hepatobiliary enzymes, hepatic steatosis, inflammation, and hepatocellular swelling can be measured to evaluate the impact of interventions. Additionally, biomarkers such as fibrosis markers (e.g., fibrosis-4 index) and non-invasive imaging techniques (e.g., transient elastography) can provide insights into the degree of fibrosis and liver stiffness [8].

Regarding the diagnosis of NAFLD, a combination of approaches is typically used. Blood tests, such as liver enzyme and liver function tests, chronic viral hepatitis tests, and lipid profiles, can help diagnose the condition and determine its severity. Imaging techniques also play a crucial role in the evaluation and management of NAFLD [26].

Identification of Advanced Fibrosis

Alanine aminotransferase (ALT) is an enzyme predominantly found in liver cells. Elevated levels of ALT in the blood indicate liver injury or inflammation, making it a valuable marker of liver damage in NAFLD. Aspartate aminotransferase (AST), another liver cell enzyme, is also used as a biomarker in NAFLD [27]. While elevated AST levels can indicate liver damage, they are less specific to liver disease compared to ALT. Gamma-glutamyl transferase (GGT) is an enzyme involved in liver and bile duct function. Elevated levels of GGT can indicate liver injury and are often used alongside other liver function tests to assess liver health in NAFLD. GGT is useful in identifying liver dysfunction and monitoring disease progression.

The fatty liver index (FLI) is a scoring system that combines several parameters, including body mass index (BMI), waist circumference, triglyceride levels, and GGT levels [27]. FLI is a noninvasive tool used to estimate the likelihood of having a fatty liver and assess the severity of hepatic steatosis. It provides a practical approach to identifying individuals at risk of NAFLD. The fibrosis-4 (FIB-4) index is a noninvasive marker used to assess the degree of liver fibrosis in NAFLD. It combines age, AST, ALT, and platelet count to estimate the fibrosis stage. The FIB-4 index is helpful in identifying patients with advanced fibrosis who may require further evaluation or intervention [26].

The enhanced liver fibrosis (ELF) test is a blood-based panel that measures specific markers associated with liver fibrosis. This panel includes hyaluronic acid, amino-terminal propeptide of type III collagen, and tissue inhibitor of metalloproteinase 1. The ELF test provides a quantitative assessment of fibrosis severity in NAFLD, aiding in the evaluation of disease progression and the efficacy of therapeutic interventions [23].

Genetic and Inflammatory Biomarkers in NAFLD/NASH

In addition to these biomarkers, inflammatory biomarkers such as C-reactive protein (CRP) and interleukin-6 (IL-6) are evaluated in NAFLD. CRP is an indicator of systemic inflammation, while IL-6 is a pro-inflammatory cytokine. These biomarkers reflect the inflammatory state associated with NAFLD and may help guide treatment strategies [27]. Adipokines and cytokines, such as adiponectin, tumor necrosis factor-alpha (TNF-α), interleukin-1 beta (IL-1β), and leptin, are also investigated as biomarkers in NAFLD. These molecules are involved in the regulation of inflammation, insulin sensitivity, and metabolic processes. Their measurement helps in understanding the complex interplay between adipose tissue, inflammation, and metabolic dysfunction in NAFLD [28].

Genetic and molecular markers, along with the gut microbiota, are pivotal in NAFLD treatment. Genetic variants like PNPLA3 rs738409 C>G, TM6SF2 E167K, and GCKR rs780094 influence hepatic lipid accumulation and disease progression. Omics-based markers provide insights into molecular profiles, while microRNAs serve as potential mechans [22]. The gut microbiota exhibits specific signatures linked to NAFLD severity. Understanding these markers enhances diagnosis, prognosis, and treatment strategies for NAFLD [1, 29].

Scoring Systems to Identify NAFLD/NASH

The non-invasive diagnosis of NAFLD includes FLI, NAFLD liver fat score (NLFS), lipid accumulation product (LAP), and novel NAFLD biomarkers. The FLI is a well-predictive algorithm that estimates hepatic steatosis. It is a preferred diagnosis technique due to the simplicity of the method. This method is based on BMI, waist circumference, serum TG, and gamma-glutamyl transferase (GCT). In this algorithm, an FLI score of <30 indicates no fatty liver, a score from 30 to 60 indicates undetermined conditions, and a score ≥60 predicts the development of hepatic steatosis.

The other diagnostic technique to predict NAFLD is the NLFS. In this technique, the liver fat content is measured with the help of proton magnetic resonance spectroscopy (H-MRS). Then it is compared to the standard hepatic steatosis fat content. This scoring system includes fasting serum insulin level, and the sensitivity level is much higher than the FLI. Statistically, the AST ratio can be noted with a sensitivity of 86% and a specificity of 71%. Another diagnostic technique widely used to identify fatty liver diseases in patients is LAP. Initially, it was developed for the US National Health and Nutrition Examination survey. It is now known as a biomarker of central obesity. This diagnostic technique separates the patients according to their fatty liver level with the help of ultrasound results. This makes it useful for checking the presence and stage of NAFLD. LAP is a powerful and easy tool to predict NAFLD in childhood. If LAP is ≥42.7, NAFLD should be suspected [28,29].

Ultrasonography

In the diagnosis and management of non-alcoholic fatty liver disease (NAFLD), various imaging techniques are employed. Conventional ultrasound (US) is commonly used as an initial imaging modality to detect fatty liver disease. It is a non-invasive technique that assesses hepatic steatosis by evaluating the liver's echogenicity [30]. Ultrasonography allows for reliable and accurate detection of moderate-severe fatty liver, compared to histology. Because of its low cost, safety, and accessibility, ultrasound is likely the imaging technique of choice for screening for fatty liver in clinical and population settings. However, it has limitations in accurately quantifying hepatic fat content and differentiating between simple steatosis and non-alcoholic steatohepatitis (NASH). Controlled attenuation parameter (CAP) is a technique that quantifies liver fat using ultrasonography, but it can be affected by obesity and has limitations in obese patients [28].

Magnetic Resonance Imaging (MRI) and Magnetic Resonance Spectroscopy (MRS)

MRI and MRS provide more precise assessments of hepatic steatosis [31]. MRI techniques, such as proton density fat fraction (PDFF) measurement, offer an accurate evaluation and can differentiate between simple steatosis and NASH. MRS, a specialized MRI technique, allows for the quantification of hepatic triglyceride content and aids in the diagnosis and monitoring of NAFLD but less widely available. MRI determines the liver fat at 5.56% in a population compared to the healthy individual [32]. PET is valuable for NAFLD treatment, detecting liver metabolic activity to assess severity and guide therapy. However, PET's limitations include limited spatial resolution, high cost, and the need for radioactive tracers. Single-photon emission computed tomography (SPECT) provides 3D images for functional assessment, evaluating perfusion and liver function in NAFLD, but has lower spatial resolution and longer acquisition times [33].

Elastography and Computed Tomography (CT)

Elastography measures liver stiffness as a non-invasive marker of fibrosis in NAFLD, though operator-dependency and limited availability may be considerations [34]. Transient elastography, known as FibroScan, is a non-invasive method used to assess liver fibrosis by measuring liver stiffness. Liver stiffness correlates with the degree of fibrosis and helps in risk stratification for NAFLD patients. FibroScan is a valuable tool for identifying individuals with advanced fibrosis who may require closer monitoring or intervention [35].

Computed tomography (CT) can identify and quantify hepatic fat content in NAFLD. It also provides additional information about liver structure and the presence of complications such as hepatocellular carcinoma (HCC). However, CT involves radiation exposure and is not typically utilized as a first-line imaging technique for NAFLD evaluation. It has limited sensitivity for mild steatosis [2], [31].

These imaging techniques, particularly MRI-based techniques like PDFF and MRS, are increasingly being used for the non-invasive assessment of NAFLD due to their ability to accurately quantify hepatic steatosis and differentiate between different stages of the disease. They offer advantages over liver biopsy, which is the current gold standard but is invasive and subject to sampling variability [33]. Table 2 summarizes the current imaging being used in NAFLD.

**Table 2:** Different imaging techniques used in NAFLD, their advantages and limitations. Adapted from Takahashi et al. [36] NAFLD: non-alcoholic fatty liver disease

Imaging Technique	Description	Advantages	Limitations
Ultrasonography	Uses high-frequency sound waves to produce images of internal organs	Non-invasive, widely available, real-time imaging, cost-effective	Limited tissue penetration, operator-dependent, image quality may be affected by body habitus or bowel gas
Computed tomography (CT)	Utilizes X-rays to create detailed cross-sectional images of the body	High-resolution, multiplanar imaging, rapid acquisition	Ionizing radiation exposure, contrast agent use may cause allergic reactions or kidney damage, limited soft tissue characterization
Magnetic resonance imaging (MRI)	Uses strong magnetic fields and radio waves to generate detailed images of the body	Excellent soft tissue contrast, multiplanar imaging, no ionizing radiation	Expensive, longer scan times, patient claustrophobia, contraindicated for patients with certain metallic implants or devices
Positron emission tomography (PET)	Combines functional and anatomical imaging by detecting radioactive tracers	Can detect metabolic activity, useful for cancer staging, whole-body imaging	Expensive, limited spatial resolution, requires injection of radioactive tracers
Single-photon emission computed tomography (SPECT)	Uses gamma cameras to detect gamma rays emitted by radioactive tracers	3D imaging, functional assessment, wide availability	Lower spatial resolution compared to PET, longer acquisition times, limited quantitative accuracy
Elastography	Measures tissue stiffness as a marker of fibrosis using ultrasound or MRI	Non-invasive, can assess liver fibrosis, real-time imaging	Operator-dependent, limited availability, may be affected by obesity or other factors
Magnetic resonance elastography (MRE)	Applies low-frequency vibrations and MRI to assess tissue stiffness	Whole liver assessment, excellent diagnostic accuracy for fibrosis	Requires MRI facility, expensive, time-consuming

Challenges and future advancements in the treatment of NAFLD

Challenges in treating NAFLD include barriers to lifestyle modifications, patient compliance with pharmacological therapies, and limited treatment options [23]. Overcoming these challenges requires addressing factors such as motivation, long-term behavioral changes, and access to resources for lifestyle interventions, as well as improving medication adherence [37]. The future of NAFLD treatment and research holds promise through personalized medicine based on genetic profiling, nanomedicine for enhanced drug delivery, modulation of the gut microbiota, and identification of novel therapeutic targets [21]. Future advancement of NAFLD therapy should focus on the mechanistic studies on cell-based and animal models and human clinical trials of exercise, as well as the combination of lifestyle intervention and pharmaceutical therapy specifically targeting main signaling pathways related to lipid metabolism, oxidative stress, and inflammation. Additionally, combination therapies that integrate lifestyle modifications, pharmacological agents, and innovative interventions can provide more effective disease management [25]. These efforts aim to advance NAFLD treatment, improve patient outcomes, and alleviate the burden of the disease.

## Conclusions

One of the most prevalent liver diseases, NAFLD still has a long way to go before it can be properly diagnosed and treated. Since there is no FDA-approved medication to treat it, the majority of care strategies rely on altering one's lifestyle and managing underlying conditions that are associated with metabolic syndrome (obesity, hypertension, diabetes, and hyperlipemia). To determine the severity and stages of NAFLD, several biomarkers are also being employed. However, more study is required to fully comprehend the pathogenesis of NAFLD, and efforts must be made to enhance therapies and diagnostics. The recent advancements in diagnosis and interventions were highlighted in this article.
